# Elevated Levels of Anti-Inflammatory Eicosanoids and Monocyte Heterogeneity in *Mycobacterium tuberculosis* Infection and Disease

**DOI:** 10.3389/fimmu.2020.579849

**Published:** 2020-11-12

**Authors:** Kristin Grotle Nore, Marthe Jøntvedt Jørgensen, Anne Ma Dyrhol-Riise, Synne Jenum, Kristian Tonby

**Affiliations:** ^1^ Faculty of Medicine, Institute of Clinical Medicine, University of Oslo, Oslo, Norway; ^2^ Department of Infectious Diseases, Oslo University Hospital, Oslo, Norway

**Keywords:** tuberculosis, eicosanoids, prostaglandins, leukotrienes, lipoxins, monocytes, T cells, host-directed therapy

## Abstract

Eicosanoids modulate both innate and adaptive immune responses in *Mycobacterium tuberculosis (Mtb)* infection and have been suggested as possible Host Directed Therapy (HDT) targets, but more knowledge of eicosanoid dynamics in *Mtb* infection is required. We investigated the levels and ratios of eicosanoid mediators and their cellular sources, monocyte subsets and CD4 T cells in Tuberculosis (TB) patients with various clinical states of *Mtb* infection. Patients consenting to prospective enrolment in a TB quality registry and biorepository, 16 with pulmonary TB (before and at-end-of treatment), 14 with extrapulmonary TB and 17 latently infected (LTBI) were included. Plasma levels of Prostaglandin E2 (PGE2), Lipoxin A4 (LXA4), and Leukotriene B4 (LTB4) were measured by enzyme-linked immunosorbent assay. Monocyte subsets and CD4 T cells and their expression of Cyclooxygenase-2 (COX-2), Prostaglandin receptor EP2 (EP2), and 5-Lipoxygenase (5-LOX) were analyzed by flow cytometry with and without Purified Protein Derivate (PPD)-stimulation. Pulmonary TB patients had elevated levels of the anti-inflammatory mediator LXA4 at diagnosis compared to LTBI (p < 0.01), while levels of PGE2 and LTB4 showed no difference between clinical states of *Mtb* infection. LTB4 was the only mediator to be reduced upon treatment (p < 0.05), along with the ratio LTB4/LXA4 (p < 0.01). Pulmonary TB patients had higher levels of total monocytes at diagnosis compared to end-of-treatment and LTBI (both p < 0.05), and a relative increase in the classical monocyte subset. All monocyte subsets had low basal expression of COX-2 and 5-LOX, which were markedly increased upon PPD stimulation. By contrast, the expression of EP2 was reduced upon stimulation. CD4 T cells expressed low basal COX-2 activity that increased modestly upon stimulation, whereas their basal expression of 5-LOX was considerable. In conclusion, the level of eicosanoids in plasma seem to vary between clinical states of *Mtb* infection. Mediators in the eicosanoid system are present in monocytes and CD4 T cells. The expression of eicosanoids in monocytes are responsive to mycobacterial stimulation independent of *Mtb* disease state, but subsets are heterogeneous with regard to eicosanoid-mediator expression. Further exploration of eicosanoid mediators as targets for HDT in TB are warranted.

## Introduction

Globally, nearly 10 million new cases of tuberculosis (TB) resulting in 1.3 million deaths, are reported annually despite intensive global strategies to fight the TB epidemic (1, 2). The sustained success of the causative agent, *Mycobacterium Tuberculosis (Mtb)*, is partly explained by complex and multifactorial mechanisms to avoid, evade and subvert host immune responses (3–7).

**Figure 1:**
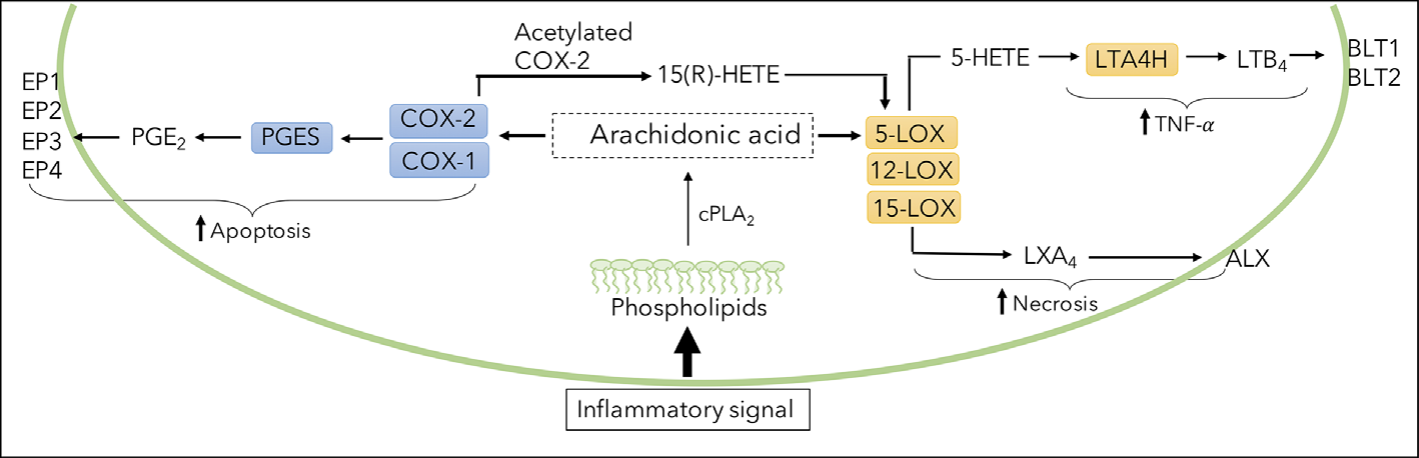
Illustration of eicosanoid biosynthesis. Formation of PGE2, LTB4, and LXA4 through breakdown of arachidonic acid (AA) by cyclooxygenase (COX) and lipoxygenase (LOX) pathways. COX enzymes (constitutive COX-1 or inducible COX-2) and downstream PGES generates PGE2 which binds to various receptors (EP1-EP4). 5-LOX-derived LTB4 is produced *via* the precursor 5-HETE, converted by enzyme LTA4H and exerts effects through receptors BLT1 and BLT2. LXA4 is derived from LOX enzymes 5,12 and 15-LOX and binds to the ALX receptor.

Host-directed therapy (HDT), aiming to enhance host immune responses and modulate *Mtb*-induced inflammation, is a possible approach to improve treatment outcomes and contribute to shorter treatment regimens (8–10). Lipid mediators of the eicosanoid family are suggested as possible HDT targets (11–14). Eicosanoid biosynthesis consists of several pathways (**Figure 1**). Breakdown of Arachidonic Acid (AA), an integral part of all cell membranes, leads to production of lipid mediators through the gate-keeping enzymes Cyclooxygenase-2 (COX-2) and 5-lipooxygenase (5-LOX). Generated through COX-2, Prostaglandin E2 (PGE2) acts through four distinct receptors, of which Prostaglandin E2 receptor 2 (EP2) seems to play a role in host susceptibility to *Mtb* infection (15, 16). In parallel, 5-LOX generates pro-inflammatory leukotrienes and anti-inflammatory lipoxins, of which Leukotriene B4 (LTB4) and Lipoxin A4 (LXA4) seem to modulate innate and adaptive immune responses by exerting pro-inflammatory or pro-resolution effects (17–19). In *Mtb* infection, the dynamics between eicosanoids may impact on protective host immune responses important for containment or progression of TB disease (6, 14, 20). Disease severity in TB has been reported to be associated with increased ratio of LXA4/LTB4 and/or reduced ratio of PGE2/LXA4, rather than changes in absolute levels of specific metabolites (21, 22). Still, it is unclear whether eicosanoids can act as biomarkers, separating latent from active TB cases or reflecting responses to TB therapy (22).

Macrophages, innate immune cells of critical importance in TB pathogenesis and disease, are major producers of eicosanoids (8, 23). Monocytes are macrophage precursors circulating in peripheral blood and are divided into different subsets; classical monocytes (CD14^++^CD16^-^), intermediate monocytes (CD14^++^, CD16^+^), and non-classical monocytes (CD14^+^CD16^++^) (24). These subsets harbor diverging biological roles in antigen presentation, phagocytosis and production of inflammatory mediators during *Mtb* infection (4) suggesting an influence on innate and adaptive host responses (25), of which effector CD4 T-cells exert crucial functions in anti-*Mtb* host defense by facilitating macrophage activation (5, 26, 27).

We hypothesized an imbalance in the levels of eicosanoids secondary to *Mtb*-induced increased COX-2 activity. We therefore explored absolute levels of lipid mediators and their ratios in plasma in different clinical states of *Mtb* infection. Further, we mapped the expression and function of relevant eicosanoid enzymes and receptors in monocyte subsets and T cells in active TB compared to latently infected individuals (LTBI). We also analyzed changes during TB treatment to better understand eicosanoid-mediated immunomodulatory mechanisms in *Mtb* infection and disease.

## Materials and Methods

### Study Population

Participants aged 18–70 years diagnosed with pulmonary TB (PTB), extrapulmonary TB (EPTB) or latent TB infection (LTBI) were recruited from the Dep. of Infectious Diseases and Dep. of Pulmonary Medicine at the Oslo University Hospital into a prospective observational cohort study (*Prognostic Immunological markers in Tuberculosis*). The TB diagnosis was based on positive sputum smears for acid fast bacilli, radiological findings, and/or *Mtb* positive cultures. MDR-TB and disseminated TB cases were excluded. All patients were treated with standard TB therapy following WHO guidelines: intensive phase of 2 months with 4 anti-TB drugs (isoniazid (INH), rifampicin (RIF), ethambutol (EMB), pyrazinamide (PZA)) and a continuation phase of 4 months with 2 anti-TB drugs. All patients received and responded to standard TB therapy for 6–12 months. LTBI were defined by positive QuantiFERON^®^-TB In Tube assay (> 0.70 IU/ml), absence of TB suspected clinical symptoms and, if relevant, negative *Mtb* cultures. Blood samples and clinical information was collected at diagnosis and, for patients with PTB disease, also at the end of anti-TB therapy after 6–12 months. To obtain an immunologically more homogenous patient cohort, exclusion criteria for all participants were HIV infection, diabetes, immunosuppressive diseases, and/or use of immunosuppressive medication.

### Eicosanoid Measurements in Plasma

Blood samples collected in EDTA vacutainers were centrifuged immediately for 20 min at 2,000 g, snap-frozen and stored at -80°C until analysis. Using a competitive parameter immunoassay, human plasma concentrations of LTB4 and PGE2 were quantified using commercial EIA kits (Cayman chemical, Ann Harbour, MI). Plasma concentrations of LXA4 were assessed using enzyme immunoassay (EIA) kits (Oxford Biomedical Research, Oxford, MI). All assays were performed according to manufacturer’s instructions. Briefly, samples underwent extraction protocols using C18-SPE Cartridges (Waters inc) or extraction using acetone precipitation, acidification and ethyl acetate extraction prior to analysis. Samples were then run in duplicates and optical density was determined at 450 or 650 nm using a Spectramax Abs plus microplate reader (Molecular devices Corporation). Lipid concentrations were calculated based on a sigmoidal standard curve using a 4-Parameter logistic fit. Inter-assay and intra-assay controls were included in all experiments.

### Cell Preparation and Stimulation

Peripheral blood mononuclear cells (PBMC) drawn on cell preparation tubes, CPT (BD Biosciences, San Jose, CA), were isolated by centrifugation and cryopreserved in 20% DMSO/80% fetal calf serum at -150 °C until analysis. Cryopreserved PBMCs were thawed in a water bath at 37°C and washed with pre-warmed RPMI 1640 (Sigma-Aldrich) media supplemented with 1% L-glutamine and 10% fetal calf serum and rested for 2h prior to cell counts and viability check (Tryphan blue microscopy). Only samples with >80% viability were included in analysis. PBMC (8x10^5^ cells) were either unstimulated or stimulated with 10 ug/ml Purified protein derivative (PPD, SSI, Denmark) and placed in a 37°C degree (with 5% CO_2_) incubator overnight (18h). Cells were subsequently stained with either a monocyte or a T cell antibody panel (See **Supplementary Tables 1** and **2**).

### Flow Cytometry Analyses

Cells were washed, stained and incubated for 30 min at 4°C. The monocyte panel consisted of Fixable Viability Dye 660, HLA-DR Alexa 700, Lineage cocktail 1 (CD3, CD20, CD56, and CD19) APC, CD14 PerCP, CD16 BV605, and EP2 PE. The T cell panel consisted of Fixable Viability Dye 660, CD3 PerCP, CD4 Alexa 700, EP2 PE. Then, cells were fixed and permeabilized according to the manufacturer’s instructions (T cell panel with eBioscience FoxP3 transcriptional factor staining kit and monocyte panel with Cytofix/cytoperm staining kit, BD Bioscience) and subsequently stained for intracellular markers with conjugated antibodies (COX-2 FITC). Unconjugated antibody for 5-LOX were stained for 30 min at 4°C, followed by 2x wash with PBS and an additional incubation with Alexa Fluor^®^ 405-conjugated secondary antibody (Abcam) was performed for another 30 min at 4°C. Flow cytometry (FACS Canto II, BD Biosciences) was performed in a blinded random order to remove any analysis bias. Fluorescence minus one (FMO) were used for gating of COX-2, EP2, 5-LOX (**Supplementary Figure 1C**). Total monocytes (HLA-DR^+^ cells gated from the monocyte cloud) were further gated into the monocyte subsets classical monocytes (CD14^++^CD16^-^), Intermediate monocytes (CD14^++^, CD16^+^) and non-classical monocytes (CD14^+^CD16^++^) (**Supplementary Figure 1A**) (24). T cells were gated from live CD3 and CD4 positive cells with measurement of COX-2, EP2 and 5-LOX (**Supplementary Figure 1B**). Data are given as frequencies or mean fluorescent intensity (MFI). Flow analysis was performed using FlowJo software (Tree Star Inc.).

### Statistical Analysis

All data are expressed with median and interquartile range (IQR). Non-parametrical statistical methods were applied. For ELISA quantification, p-values were calculated using the Kruskal-Wallis test with Dunn’s post-hoc for multiple comparisons and Wilcoxon signed rank test was used for paired samples. For flow cytometry data, Mann-Whitney U test was used for groupwise comparison of unpaired data and Wilcoxon for matched pair test by using Graphpad Prism version 8.0 (Graphpad Software, LA Jolla, CA) and SPSS (IBM).

### Ethical Considerations

The study was approved by the Regional Committees for Ethics in Medical Research (REK-Sør-Øst 2016/2123). Biobank samples were collected and stored in the “Research Biobank Infectious Diseases” (“Forskningsbiobank Infeksjonssykdommer” (REK 1.2006.181-S-0885, SHDNR. 09/513), Department of Infectious Diseases, OUS, Ullevål. Written informed consent was obtained from all participants before inclusion.

## Results

### Study Participants Characteristics

Thirty patients with TB disease (14 patients with EPTB and 16 patients with PTB) and 17 patients with LTBI, were included. Demographic and clinical variables of *Mtb* infected patients are listed in **Table 1**.

**Table 1:** Demographic and clinical variables in patients with Mtb infection and disease.

	EPTB n = 14	PTB N = 16	LTBI N = 17
Gender, male (n, %)	9 (64)	13 (81)	6 (35)
Age (median, range)	30 (18–40)	32 (18–64)	29 (20–54)
Ethnicity (n, %)			
Caucasian	3 (21)	6 (37)	0 (0)
African	8 (57)	3 (19)	5 (29)
Asian	2 (14)	6 (37)	9 (52)
Unknown	1 (7)	1 (6)	3 (18)
Previous TB treatment (n, %)			
Yes	1 (7)	2 (13)	0 (0)
Unknown	2 (14)	1 (6)	0 (0)
Confirmed *Mtb* complex in culture/PCR (%)			
Yes	8 (57)	13 (81)	0 (0)
Resistance (%)			
Monoresistant TB^1^	5 (36)	2 (13)	
No of TB localizations (%)			
1	12 (86)	13 (81)	
2	2 (14)	3 (19)	
Low: High symptom score^2^	7:7	5:11	16:1
QuantiFERON-TB Gold (positive: negative: no data)	12:0:2	9:3:4	17:0:0
ESR^3^ at baseline (mm/hour, range)	32 (5–82)	35 (3–109)	21 (13–40)
CRP^3^ at baseline (mg/L, range)	17 (1–89)	19 (0.6–97)	3 (0.7–10)

^1^Monoresistance is defined as resistance to one first-line anti-TB drug only.

^2^High symptom score is defined as 2 or more of the following symptoms: Fever (>38.0°), weight loss, cough, lymphadenopathy, night sweat. Low symptom score is defined as one of the symptoms listed or asymptomatic.

^3^ESR, erythrocyte sedimentation rate; CRP, C- reactive protein.

### Levels of Plasma Eicosanoids During Different Stages of *Mtb* Infection

**Figure 2:**
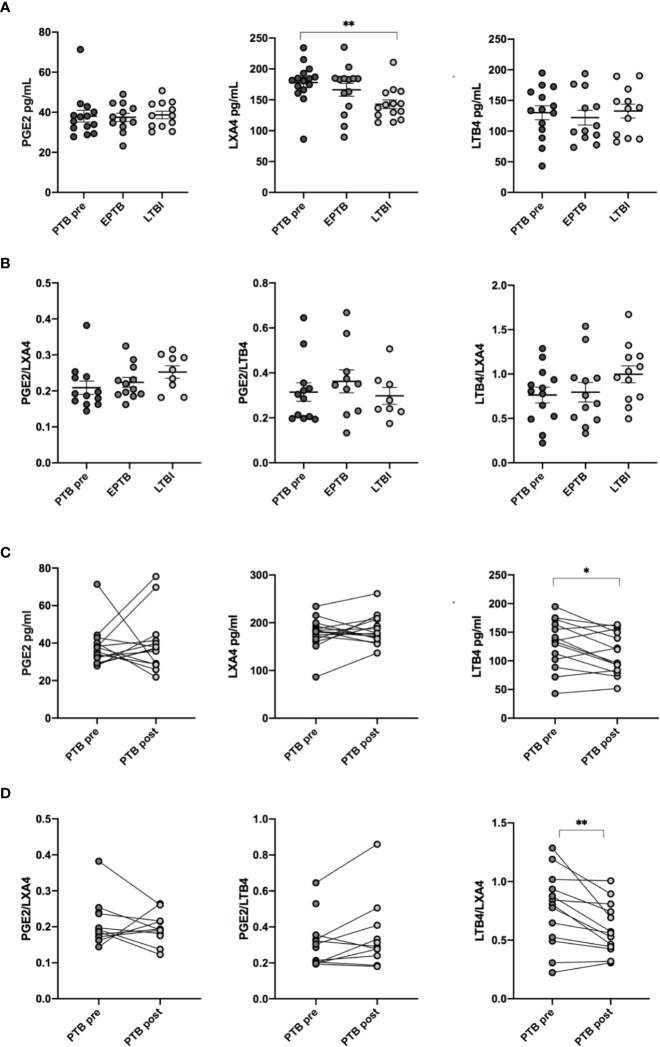
Concentration and ratio of LXA4, PGE2 and LTB4 in plasma. **(A)** Concentrations of eicosanoid metabolites in pg/ml in plasma measured by ELISA at different stages of *Mtb* infection: EPTB (N = 14), PTB at time of diagnosis (PTB pre, N = 16), and LTBI (N = 17) **(B)** Ratio of eicosanoid metabolites in plasma in different stages of *Mtb* infection. **(C)** Levels of eicosanoid metabolites before (PTB pre, N = 16) and after treatment (PTB post, N = 16). **(D)** Ratios of eicosanoids before and after treatment. Data presented as median with interquartile ranges. P-values were calculated using the Kruskal-Wallis test with Dunn’s post-hoc for multiple comparisons and Wilcoxon signed rank test was used for paired samples. Statistical significance represented by asterisk: ns, not significant; *p < 0.05; **p < 0.01.

Plasma levels of PGE2, LXA4, and LTB4 were analyzed at diagnosis of TB disease (PTB and EPTB) and compared to LTBI. No significant differences in either PGE2 or LTB4 levels between the groups were observed (**Figure 2A**). In contrast, concentrations of the anti-inflammatory LXA4 were elevated in PTB compared to LTBI (p < 0.01). Concentrations of PTB and EPTB were comparable. Next, the effects of eicosanoids may depend on their relative contribution rather than absolute levels. We therefore investigated the following eicosanoid ratios: PGE2:LXA4, PGE2:LBT4 and LTB4:LXA4 (**Figure 2B**). However, there were no significant differences in either PGE2:LXA4, PGE2:LTB4 or LTB4/LXA4 ratios between the various groups at baseline.

During TB treatment, LTB4 levels decreased significantly in PTB patients (p < 0.05), whereas LXA4 and PGE2 levels did not change (**Figure 2C**). LTB4/LXA4 ratio was reduced during treatment (p < 0.01), while PGE2/LXA4 and PGE2/LTB4 ratio showed no difference (**Figure 2D**).

Eicosanoid balance may be affected by host genomic background and genetic polymorphism (28), we therefore investigated levels and ratios of eicosanoids by stratifying patients according to ethnicity (Caucasian, African, and Asian), but no differences were detected (**Supplementary Figure 2**).

### Monocytes, Monocyte Subsets, and Expression of COX-2, EP2, and 5-LOX

**Figure 3:**
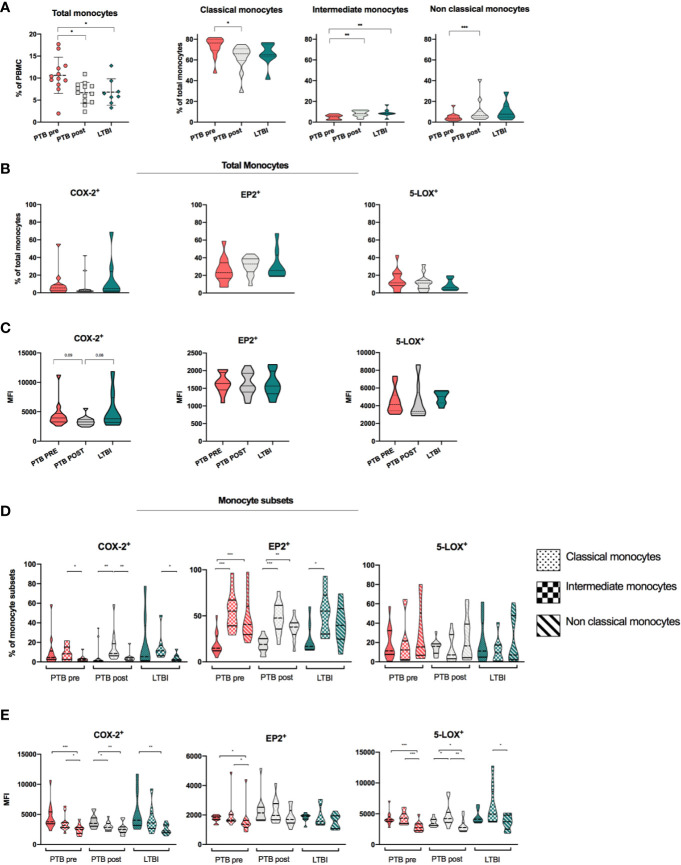
Expression of monocytes, monocyte subsets and eicosanoid expression in stages of *Mtb* infection. **(A)** Distribution and frequencies of monocytes and monocyte subsets in different clinical stages of *Mtb* infection. PTB at time of diagnosis (PTB pre, pink, N = 13), PTB at end- of- treatment (PTB post, grey, N = 13), and LTBI (green, n = 8). Monocyte subsets were defined as classical monocytes (CD14^++^CD16^-^), Intermediate monocytes (CD14^++^CD16^+^) and non-classical monocytes (CD14^+^CD16^++^) (Gating strategy **Supplementary Figure 1A**) (29). **(B)** Frequencies of COX-2, EP2, and 5-LOX in unstimulated samples in total monocytes. **(C)** Mean fluorescence intensity (MFI) of COX-2, EP2, and 5-LOX in unstimulated samples in total monocytes. Frequencies **(D)** and MFI **(E)** of COX-2, EP2, and 5-LOX in unstimulated classical, intermediate, and non-classical monocyte subsets, presented by fill pattern, stratified by patient group (color). Mann-Whitney test was used for unpaired samples, Wilcoxon signed rank test used for paired samples. Statistical significance represented by asterisk: *p < 0.05; **p < 0.01; ***p < 0.001.

We found increased frequencies of total monocytes in PTB at diagnoses compared to LTBI (11 vs 6.5%, p < 0.05) and with a significant reduction at end-of-treatment (p < 0.05) (**Figure 3A**). The distribution of monocyte subsets revealed higher frequencies of CD14^++^CD16^-^ classical monocytes (CM) in PTB at diagnosis compared to LTBI, with a significant reduction during treatment (p <0.05, median 76% in PTB at time of diagnosis vs median 66% in PTB at end-of –treatment). Frequencies of both CD14^++^CD16^+^ intermediate monocytes (IM) and CD14^+^CD16^++^non-classical monocytes (NCM) were low in PTB at diagnosis, however with a significant increase in both IM and NCM at end-of-treatment (p <0.01 and p <0.001, respectively). LTBI had significantly higher frequencies of IM compared to PTB at diagnosis (p < 0.01) (**Figure 3A**).

We further analyzed the expression of COX-2, 5-LOX, and EP2 in total monocytes in different states of *Mtb* infection (**Figure 3B**). %COX-2 expression was low and comparable in PTB at diagnosis (5.5%) and LTBI (4.8%), and with no significant changes during TB treatment (PTB post: 2.5%). Levels of EP2 were higher (PTB Pre: 23.1%, PTB post: 32.9%, and LTBI 25.6%), while levels of 5-LOX were comparable to COX-2 (PTB Pre: 11.55%, PTB post: 11.2%, and LTBI 5.9%), with no significant differences between patient groups (**Figure 3B**). When comparing MFI levels, PTB at diagnosis and LTBI showed a trend of increased levels of COX-2 compared to PTB at end-of treatment (p = 0.09 and p = 0.08, respectively), while there were no differences in levels of EP2 and 5-LOX (**Figure 3C**).

As expression of eicosanoid enzymes may be unevenly distributed in different monocyte subsets, we further explored the eicosanoid expression between the CM, IM, NCM monocyte subsets in the different stages of *Mtb* infection. Overall, as shown for total monocytes, COX-2 expressing cells were low in all monocyte subsets, but significantly higher in IM subsets (8.3%) compared to NCM (2.3%) (P < 0.05) in PTB at diagnosis (**Figure 3D**). Expression of EP2 was modest in CM in all patient groups (PTB pre: 14.9, PTB post: 19.0, LTBI: 16.8). Interestingly, levels of EP2 were significantly elevated in both the IM and NCM subsets compared to CM in PTB at diagnosis and at end-of treatment (p < 0.001 and p < 0.01) (**Figure 3D**). There were no differences in frequencies of COX-2, EP2 and 5-LOX-expressing cells between patient groups (**Supplementary Figure 3**). When investigating MFI levels, COX-2 levels were higher in CM compared to NCM in all groups (p <0.001, p <0.01, and p <0.01 for PTB pre, PTB post, and LTBI), while levels of 5-LOX were higher in CM and IM subsets compared to NCM in PTB at diagnosis and at end-of treatment (p < 0.001 and p < 0.05) (**Figure 3E**).

### Dynamics of COX-2, EP2, and 5-LOX in Monocytes Upon *In Vitro* PPD Stimulation

**Figure 4:**
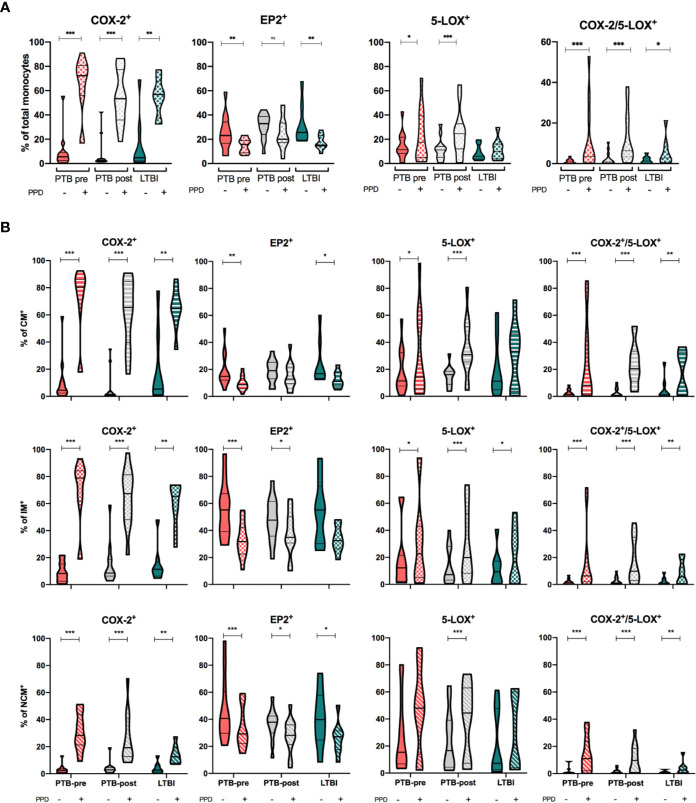
Eicosanoid expression in unstimulated and PPD-stimulated monocytes. **(A)** Expression of COX-2, EP2, 5-LOX and double positive (COX-2/5-LOX) expression in HLA-DR^+^ Total monocytes in unstimulated (filled bar) and stimulated (hatched bar) samples, stratified by patient group: PTB-pre (PTB at diagnosis, pink, n = 13), PTB post (PTB at end- of- treatment, grey, n = 13), and LTBI (latent TB, green, n = 8). **(B)** Expression of COX-2, EP2, 5-LOX, and double positive (COX-2/5-LOX) expression in unstimulated (filled bar) and stimulated (hatched bar) Classical (CD14^++^CD16^-^), Intermediate (CD14^++^CD16^+^) and non-classical monocytes (CD14^+^CD16^++^) stratified by patient group (color). Gating strategy is shown in **Supplementary Figure 1**. Mann-Whitney test was used for unpaired samples, Wilcoxon signed rank test used for paired samples. Statistical significance represented by asterisk: *p < 0.05; **p < 0.01; ***p < 0.001.

We then investigated how PPD-stimulation affected the expression of COX-2, 5-LOX and EP2 in monocytes and monocyte subsets. In total monocytes, COX-2-expression was significantly increased by PPD (p < 0.01), while EP2-expression was significantly downregulated for all groups (p < 0.01) except in PTB at end-of- treatment (**Figure 4A** and **Supplementary Figure 4**). 5-LOX mimicked COX-2 responses and was significantly up-regulated in PTB at diagnosis and at end- of- treatment (p < 0.05 and p < 0.001) (**Figure 4A** and **Supplementary Figure 4**). Next, we investigated co-expression of COX-2 and 5-LOX in unstimulated and stimulated monocytes. Frequency of double positive monocytes were low in all groups at baseline, but with significant upregulation by PPD in all groups (p <0.001, p<0.001, p <0.05 for PTB pre, PTB post, and LTBI, respectively) (**Figure 4A**).

Within the various monocyte subsets, PPD stimulation induced a significant increase in COX-2 expression regardless of group (p < 0.01), however most pronounced for CM and IM monocytes (p < 0.05) (**Figure 4B** and **Supplementary Figure 4**). Downregulation of EP2 upon PPD stimulation was seen for all monocyte subsets in all patient groups, however most pronounced for the intermediate monocytes in PTB at diagnosis (p < 0.001) (**Figure 4B** and **Supplementary Figure 4**). Although 5-LOX was upregulated upon PPD stimulation in several of the monocyte subsets, there were no differences in induced 5-LOX expression between subsets in any of the clinical states of *Mtb* infection (**Figure 4B** and **Supplementary Figure 4**). Co-expression of COX-2/5-LOX was significantly upregulated in all subsets by PPD (p = 0.001, p = 0.001, and p = 0.01 in PTB pre, PTB post, and LTBI respectively) (**Figure 4B** and **Supplementary Figure 4**).

**Figure 5:**
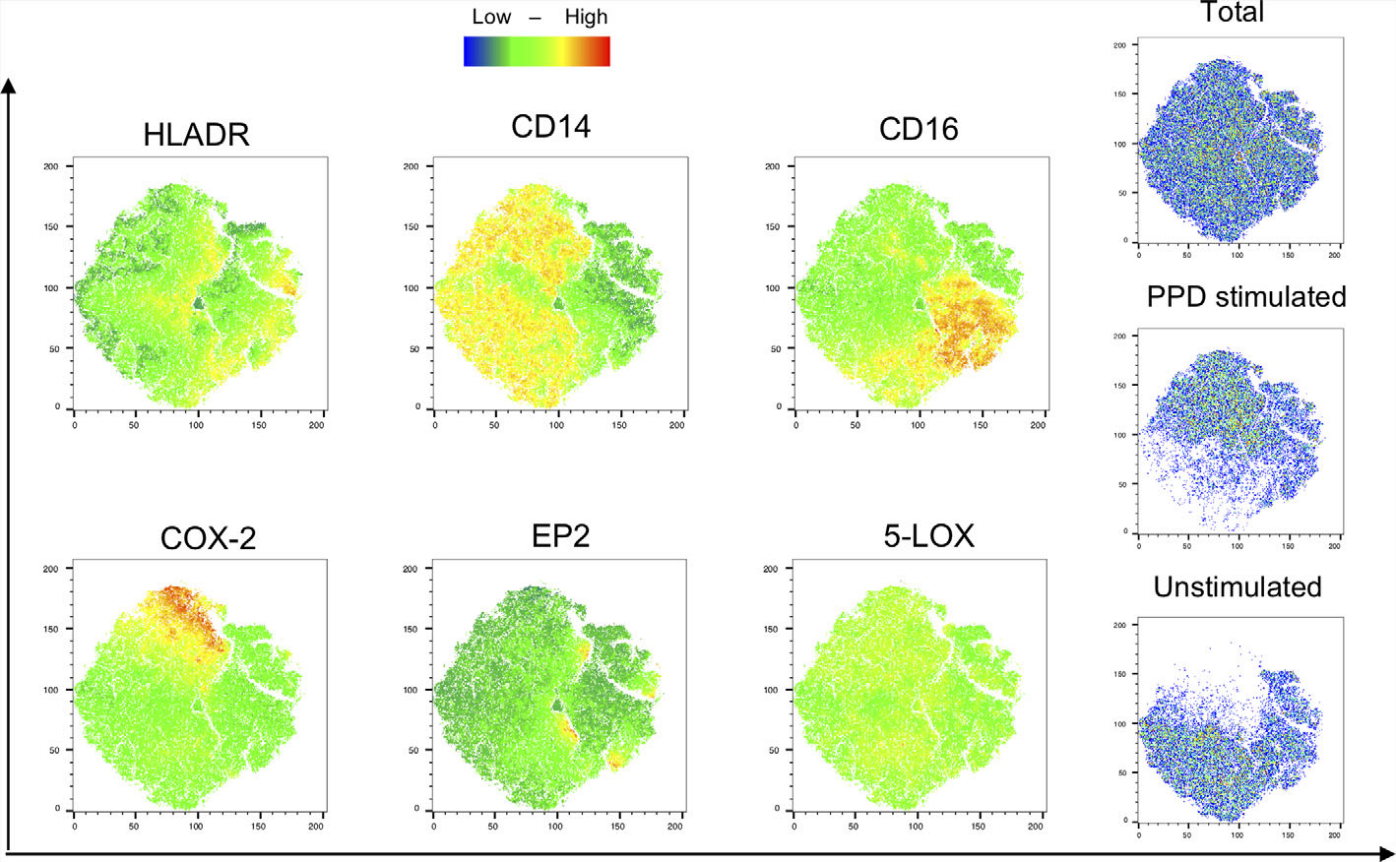
Visualization of phenotypic changes with T-sne. Visualization of monocyte-related markers (HLA-DR, CD14, CD16) and the eicosanoid enzymes/proteins (COX-2, EP2, and 5-LOX) were analyzed with an unbiased approach using t-Distributed Stochastic Neighbor Embedding (tSNE) algorithm. Unstimulated and PPD-stimulated samples from 1 patient with PTB at diagnosis were concatenated and downsampled to 3*10^5^ cells/group. Color scale denotes low (blue) to high (red) density of the markers.

Next, we evaluated the monocyte population in unstimulated and stimulated samples from PTB patients at diagnosis by the t-distributed stochatic neighbouring embedding algorithm (t-SNE) for unbiased visualization of COX-2, EP2, and 5-LOX-expressing subpopulations (**Figure 5**). This showed that EP2 is expressed by small clusters of cells in both unstimulated and stimulated regions, while 5-LOX was widely expressed throughout the monocyte populations. COX-2 is highly expressed in stimulated regions that also expressed high levels of CD14, and low levels of CD16. HLA-DR and CD14 were found in both stimulated and unstimulated regions of the monocyte population, while CD16 is mostly present in unstimulated samples (**Figure 5**).

### Expression of Eicosanoid Enzymes in CD4 T Cells

**Figure 6:**
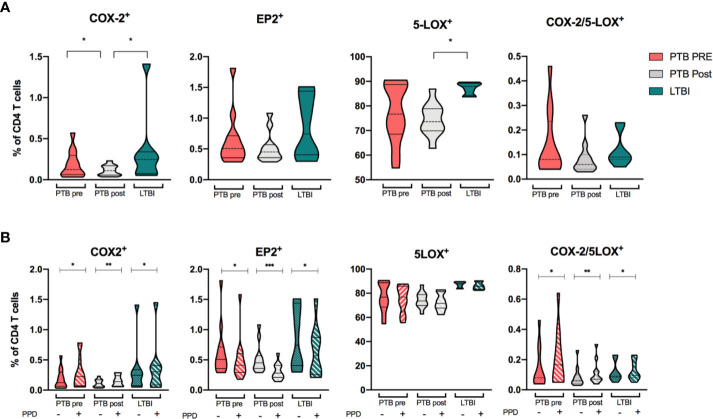
Eicosanoid expression in CD4 T cells. **(A)** COX-2, EP2, 5-LOX, and double positive (COX-2/5-LOX) expression in unstimulated samples from patients with PTB at diagnosis (pink, n = 13), PTB at end-of-treatment (grey, n = 13), and LTBI (green, n = 8) in CD4 T cells. **(B)** Unstimulated (filled bar) and PPD-induced expression (hatched bar) of COX-2, EP2, 5-LOX, and COX-2/5-LOX in CD4 T cells. Wilcoxon matched-pairs signed rank test was used for paired data, Mann-Whitney for unpaired group comparisons. Statistical significance represented by asterisk: ns, not significant; *p < 0.05; **p < 0.01; ***p < 0.001.

Since effector CD4 T-cells are important in anti-*Mtb* host defense we explored the relevance of this cell subset in eicosanoid biosynthesis by analyzing their expression of COX-2, EP2, and 5-LOX. Basal levels of COX-2 and EP2 expression in CD4 T cells were generally low for both PTB at diagnosis and LTBI, with even lower frequencies in the PTB group at end-of-treatment (p < 0.05). In contrast, 5-LOX–expression was high in all clinical groups, especially in LTBI (**Figure 6A**). Basal co-expression of COX-2/5-LOX was low in all groups with no significant differences between patient groups (**Figure 6A**). Next, we investigated the capacity of T cells to induce eicosanoid enzymes upon PPD-stimulation (**Figure 6B** and **Supplementary Figure 5**). For all groups, COX-2 was modestly, but significantly induced (p <0.05, p <0.01 and p <0.05 for PTB pre, PTB post and LTBI, respectively), while EP2 expression decreased in all groups (p <0.05, p <0.001, and p <0.05). PPD-stimulation did not affect 5-LOX expression, but double positive COX-2/5-LOX T cells were significantly induced by stimulation (p <0.05, p <0.01, and p <0.05 for PTB pre, PTB post, and LTBI, respectively) (**Figure 6B**).

## Discussion

Several studies have emphasized the role of eicosanoids and lipid mediators in TB pathogenesis (30–32). We hypothesized that the AA-derived metabolites PGE2, LXA4, and LTB4 reflects the ongoing inflammation and resolution processes orchestrated by eicosanoids in TB. We report absolute levels and ratios of eicosanoids, and the expression of key enzymes and proteins within the eicosanoid network in monocyte subsets and CD4 T cells, in different states of *Mtb* infection of interest to future HDT strategies.

We show that patients with TB disease have elevated plasma levels of the anti-inflammatory mediator LXA4 compared to LTBI. Levels of LTB4 decreased during treatment, whereas LXA4-levels remained unchanged and consequently the ratio of LTB4/LXA4 was lower after anti-TB therapy. Further, patients with TB disease had higher frequencies of predominately classical monocytes. The monocyte subsets in all patient groups expressed low basal levels of eicosanoid proteins, but while COX-2 and 5-LOX increased upon PPD stimulation, EP2 was reduced. Our data demonstrates dynamic changes and an imbalance in eicosanoids in TB that is not fully restored after treatment.

### Pulmonary TB Patients Exhibit a Relative Increase in Anti-Inflammatory Eicosanoids

We report that PTB patients exhibit a relative increase in the anti-inflammatory eicosanoid LXA4 compared to LTBI at diagnosis, which extends beyond treatment. This finding has also been reported in previous studies and may propose a role for LXA4 as a potential biomarker for TB disease (21, 22). Moreover, LXA4 has been linked to increased bacterial survival due to abrogated host immunity caused by promoted necrosis of infected macrophages and suppressed production of TNF-α (14, 18), suggesting a role for LXA4 in TB progression. By contrast, the pro-inflammatory mediator PGE2 has been suggested to confer protective responses during TB disease, by acting in a cross-regulatory network with IL-1 and promoting apoptosis of infected macrophages (20). Strict classification of PGE2 with regard to anti- or pro-inflammatory capacity is however less evident as PGE2 has been shown to harbor both pro- and anti-inflammatory effects depending on concentration and timing at local sites of infection (33–35). In contrast to other reports showing increased levels of PGE2 in TB disease (20–22), we observed no difference in PGE2-levels or PGE2:LXA4 ratio between patient groups. However, we observed reduced ratios of LTB4:LXA4 upon treatment, likely reflecting the reduced levels of LTB4 while LXA4 remained unchanged. In TB, an optimal balance of LTB4 is considered preferable, as high levels of this mediator tips the balance towards excess production of TNF-α, macrophage necrosis and enhanced growth of *Mtb* (18). Our data with no significant differences in levels of LXA4 and PGE2 during treatment of PTB, may be linked to treatment induced inflammatory imprinting of eicosanoid profiles, as shown in other studies (36, 37): Even though the inciting stimulus is cleared, the production of anti-inflammatory mediators may be extended and occur months after initial exposure (33, 37). Hence, future studies on dynamics of eicosanoids in *Mtb* infection should preferably secure samples 6–12 months after finalized TB treatment.

### Monocyte Heterogeneity and Eicosanoid Signaling in *Mtb* Infection

We report increased frequencies of total monocytes in PTB at diagnosis compared to end-of-treatment and LTBI. Classical monocytes constituted the major subset regardless of clinical state, while frequencies of intermediate monocytes and non-classical monocytes were low but increase moderately during treatment. Monocytes are reported to expand during TB progression (38), and our data suggests that this expansion is mostly due to increased frequency of classical monocytes. However data are conflicting, and some report an association between CD16^+^ expansion (i.e. intermediate and non-classical monocytes) and disease severity (25, 38–40), whilst others have shown higher levels of CD16^+^ monocytes in LTBI patients (41). Notably, our study did not assess severity within PTB patients.

Although functions of monocyte subsets in *Mtb* infection are reported by others (42), evidence is scarce concerning their contribution in eicosanoid regulation. We report low basal expression of COX-2 in all monocyte subsets, but significantly higher expression in intermediate monocytes, possibly indicating higher production of PGE2 in this subset. Other studies have shown that intermediate monocytes produce high levels of TNF-α, IL-6 and IL-1, but reduced levels of IL-10 (42–44). Thus, the finding that intermediate monocytes express high frequencies of COX-2, as well as elevated expression of EP2 compared to classical subsets, may support the notion that these cells are important producers of inflammatory mediators in general. Further, high levels of EP2 in intermediate and non-classical monocytes suggests that these cells are more susceptible to PGE2 signaling, compared to classical monocytes, indicating a pivotal role for intermediate and non-classical monocytes in eicosanoid signaling.

COX-2 and 5-LOX in total monocytes and monocyte subsets are clearly increased upon mycobacterial stimulation, while expression of EP2 is reduced. As classical monocytes and intermediate monocytes are considered superior phagocytes and important for secreting pro-inflammatory cytokines, it is not surprising that these subsets show elevated levels of COX-2 upon stimulation (24, 45). However, the finding that 5-LOX mimics the upregulated response of COX-2, indicates that mycobacterial antigens promote parallel induction of both enzymes also reflected in the increased levels of the anti-inflammatory LXA4 in PTB patients, as shown in our study. Our results therefore support suggestions of mycobacterial antigens manipulating the host response toward further bacterial spread through eicosanoid imbalance resulting from 5-LOX-induction and generation of LXA4 (14). Further, we found that basal levels of monocytes co-expressing COX-2 and 5-LOX were low, albeit with a significant upregulation by PPD. This strengthens our findings that monocyte subsets differ in their contribution to eicosanoid production. Reduced EP2 expression upon PPD stimulation may indicate a negative feedback loop in *Mtb* infection resulting in reduced PGE2-effects, as suggested by others describing a strong regulatory connection between COX-2 and EP2 in PGE2 synthesis (46). EP2 is reported to be distinctly upregulated upon LPS stimulation (47), and is normally not internalized or desensitized upon stimulation (48, 49). Thus, our findings of a marked reduction in EP2 receptor upon PPD stimulation raise questions regarding the potential for COX inhibitors as HDT in TB as the natural increase in EP2 expression observed in other bacterial infections seems to be abrogated by *Mtb*.

### Eicosanoid Expression in Cells of the Adaptive Immune Response

In accordance with others (50, 51), we demonstrate that eicosanoid enzymes are also expressed by CD4 T cells. The COX-2-gene is transcriptionally upregulated in human T cells during T cell receptor signaling *in vitro* (48). Although CD4 T cells express low basal COX-2 activity, expression is modestly induced upon stimulation with PPD, indicating a functional role in response to *Mtb* infection with CD4 T cells capable of PGE2 production. Surprisingly, CD4 T cells show very high levels of basal 5-LOX activity, which is seemingly unaffected by PPD stimulation. Although it has been shown that 5-LOX is expressed by T cells (51), to our knowledge, this has not previously been shown in the context of TB. 5-LOX expression in T cells may possibly suggest a capacity for CD4 T cells to produce the anti-inflammatory mediator LXA4 or the pro-inflammatory LTB4. The unexpectedly high levels of 5-LOX in CD4 T cells should be explored in future studies.

There are some limitations to our study. First, a small number of patients give reduced power in statistical calculations and small differences may not have been detected. Further, we have not analyzed genes important in eicosanoid biosynthesis, e.g *lta4h* locus polymorphism as shown in previous reports (18). Future studies on eicosanoid enzyme and receptor polymorphism, as well as expression of other key markers in eicosanoid biosynthesis involved in TB pathogenesis (e.g. LTA4H, EP1-3, 15-LOX, and PTGES), are warranted. Changes and dynamics in peripheral blood may not reflect immune responses at local sites of *Mtb* infection. Thus, our study design limits our possibility to conclude on causative factors for TB progression, but still highlights important aspects of eicosanoid biosynthesis in *Mtb* infection.

### Concluding Remarks

In this study, we show that levels of eicosanoids in plasma vary between clinical states of *Mtb* infection with an increased anti-inflammatory profile in active TB disease. Imbalance of eicosanoids extends beyond treatment. Monocytes and CD4 T cells express mediators involved in eicosanoid signaling, but monocyte subgroups differ with regard to their responsiveness and contribution to eicosanoid mediators. PPD-stimulation induced changes in eicosanoid mediators that could represent a *Mtb* strategy to divert host immune responses, but this need further investigation. Causative or not, monocyte heterogeneity is likely to impact on TB pathogenesis by influencing the balance of mediators of the eicosanoid pathway, but more mechanistic in-depth studies are warranted to understand the full network interaction. Therefore, our data support future studies exploring the role of eicosanoid mediators in immunopathogenesis in *Mtb* infection and as potential targets for HDT strategies in TB.

## Data Availability Statement

The raw data supporting the conclusions of this article will be made available by the authors, without undue reservation.

## Ethics Statement

The studies involving human participants were reviewed and approved by the Regional Committees for Ethics in Medical Research (REK-Sør-Øst 2016/2123). Biobank samples were collected and stored in the “Research Biobank Infectious Diseases” (“Forskningsbiobank Infeksjonssykdommer” (REK 1.2006.181-S-0885, SHDNR. 09/513), Department of Infectious Diseases, OUS, Ullevål. The ethics committee waived the requirement of written informed consent for participation.

## Author Contributions

Conceived and designed the experiments: KN, MJ, KT, SJ, and AD-R. Recruited patients and collected clinical data: SJ, AD-R, and KT. Data acquisition: KN and MJ. Analyzed the data: KN, MJ, SJ, AD-R, and KT. Drafted and reviewed the manuscript: KN, MJ, SJ, AD-R, and KT. All authors contributed to the article and approved the submitted version.

## Funding

The study was funded by The Research Council of Norway through the Medical Student Research Program at the University of Oslo, and the Department of Infectious diseases, Oslo University Hospital.

## Conflict of Interest

The authors declare that the research was conducted in the absence of any commercial or financial relationships that could be construed as a potential conflict of interest.

## Abbreviations

AA, Arachidonic acid; CM, Classical monocytes; COX-2, Cyclooxygenase 2; EP2, Prostaglandin E2 receptor 2; HDT, Host-directed therapy; IFN-γ, Interferon-γ; IM, Intermediate monocytes; IL-1, Interleukin 1; LTB4, Leukotriene B4; LXA4, Lipoxin A4; *Mtb*, Mycobacterium tuberculosis; NCM, Non-classical monocytes; PGE2, Prostaglandin E2; PBMC, Peripheral blood mononuclear cells; TB, Tuberculosis; TNF-α, tumor necrosis factor α; 5-LOX, 5-lipoxygenase.
