# Supplementary figures and images for: Elevated Levels of Anti-Inflammatory Eicosanoids and Monocyte Heterogeneity in Mycobacterium tuberculosis Infection and Disease
Source: Front Immunol. 2020 Nov 12;11:579849. doi: 10.3389/fimmu.2020.579849 (PMC7693556; doi:10.3389/fimmu.2020.579849)

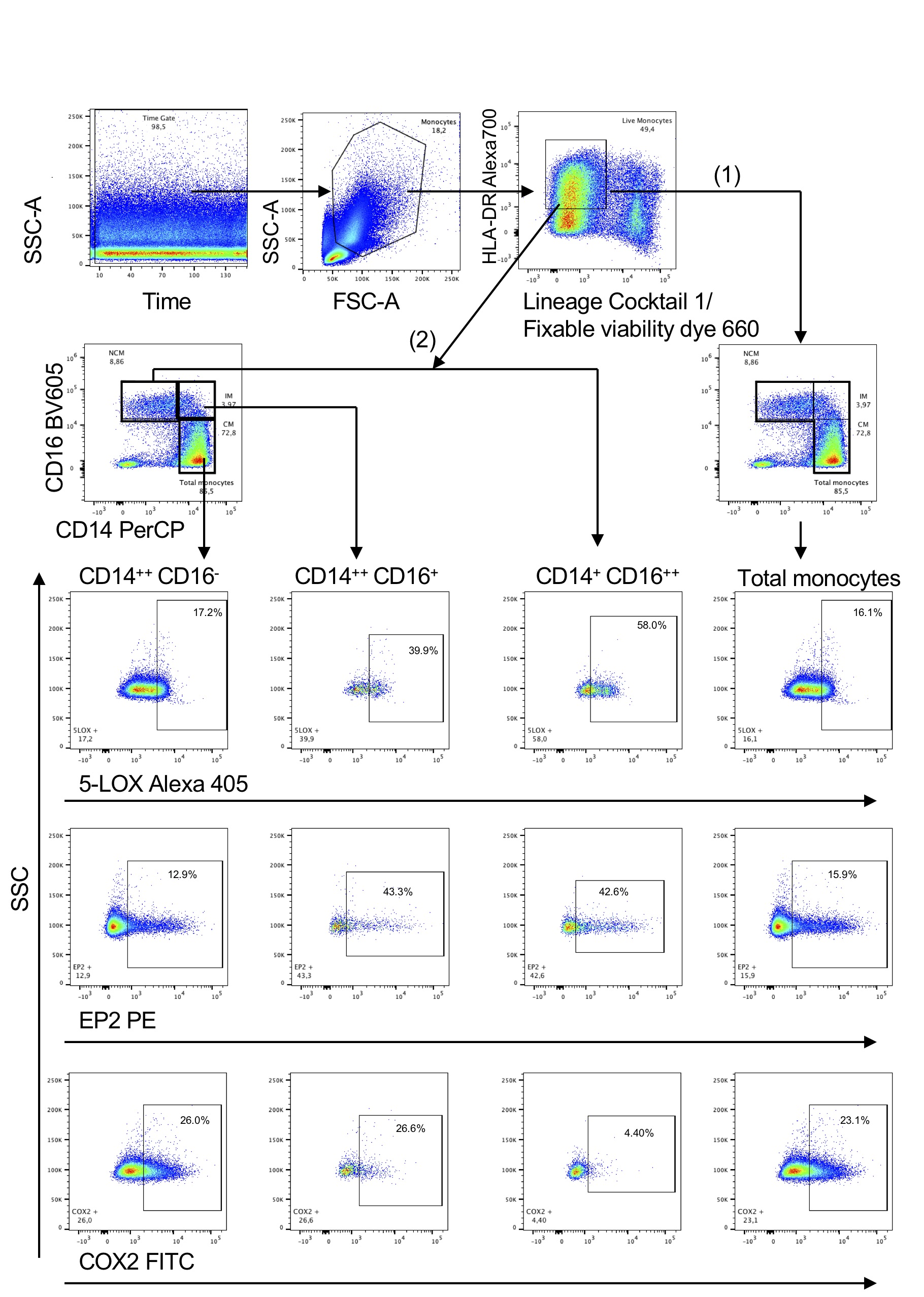

Supplement: Supplementary file 1 [file Image_1.tiff]

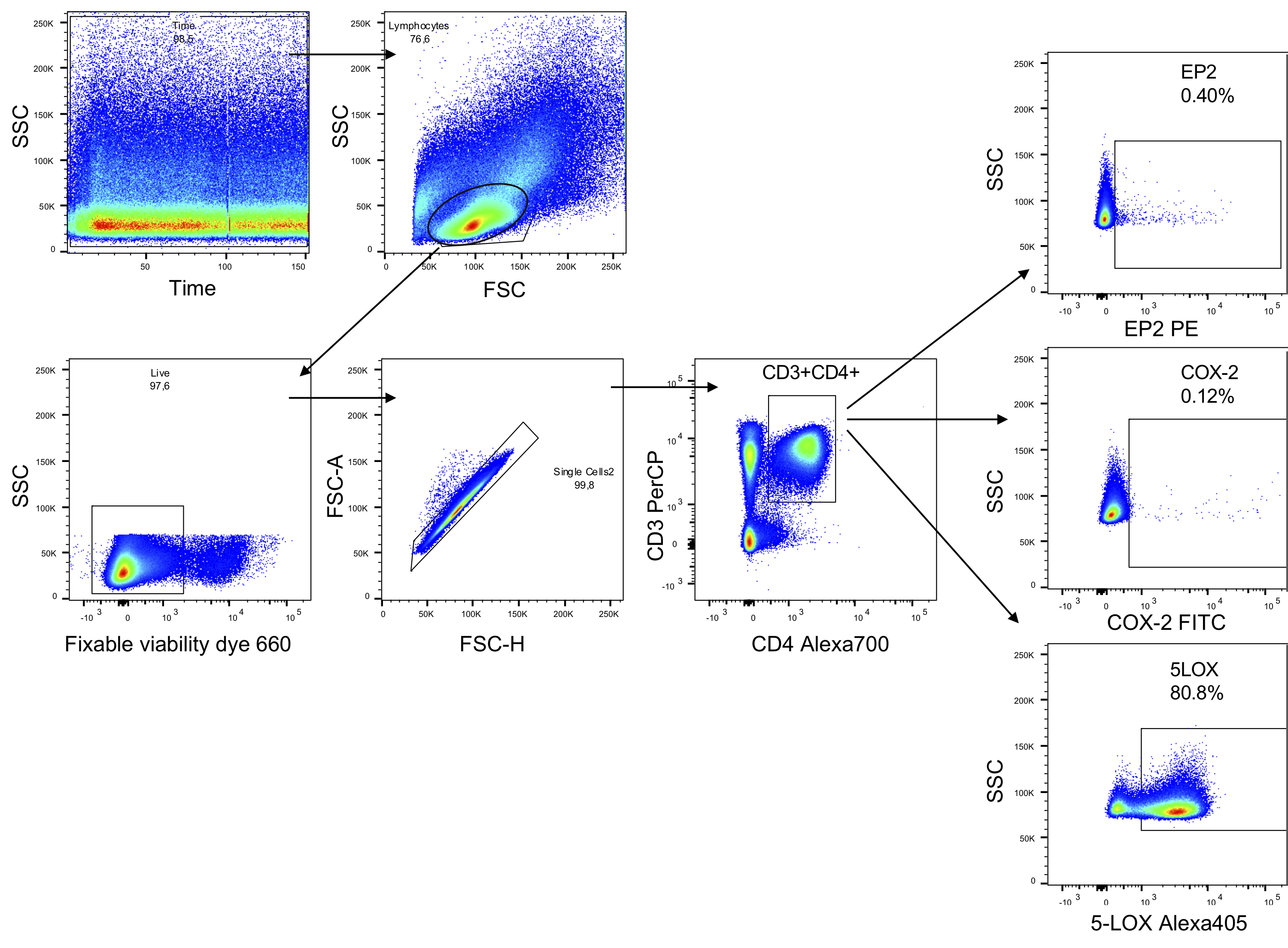

Supplement: Supplementary file 2 [file Image_2.tiff]

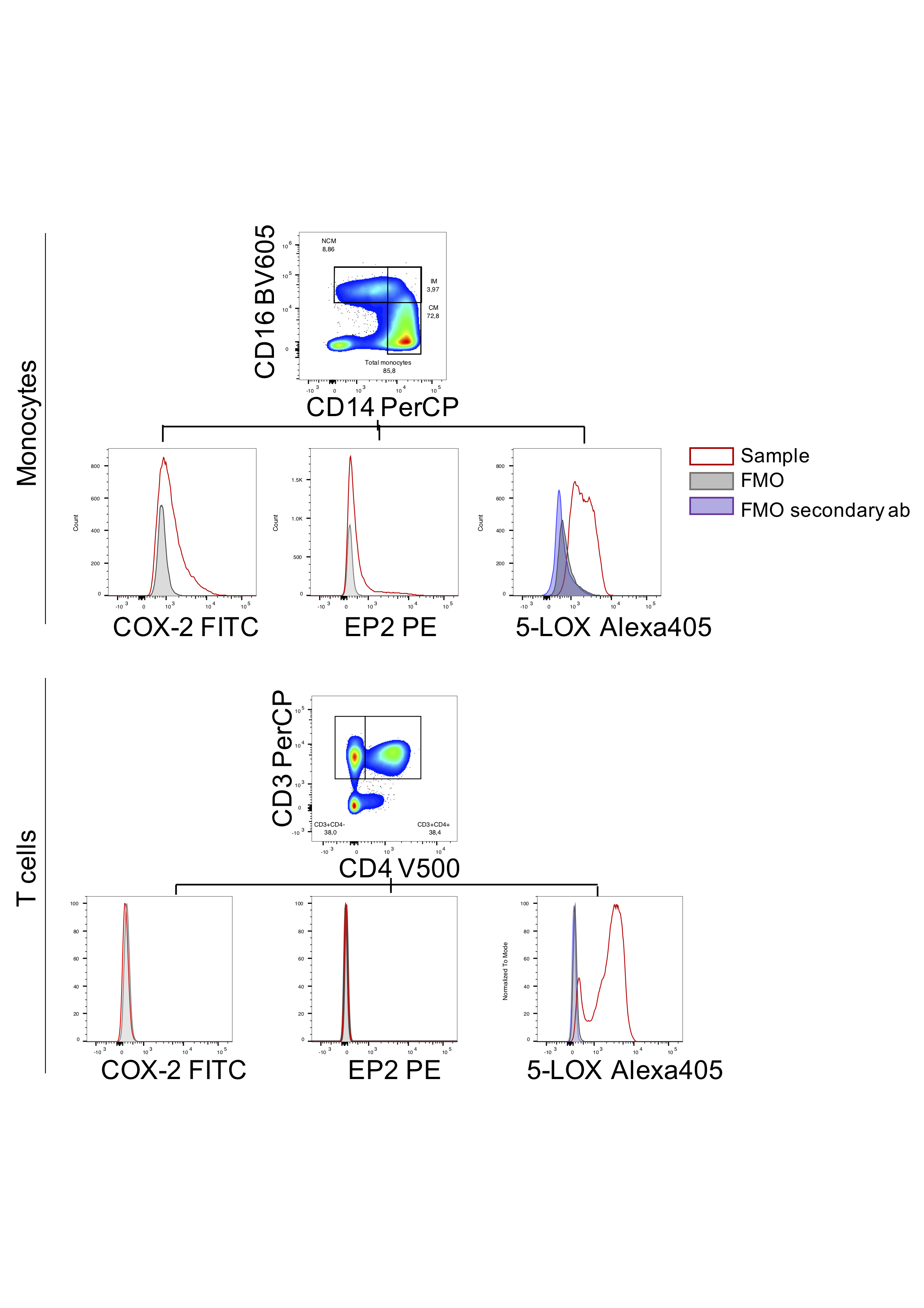

Supplement: Supplementary file 3 [file Image_3.tiff]

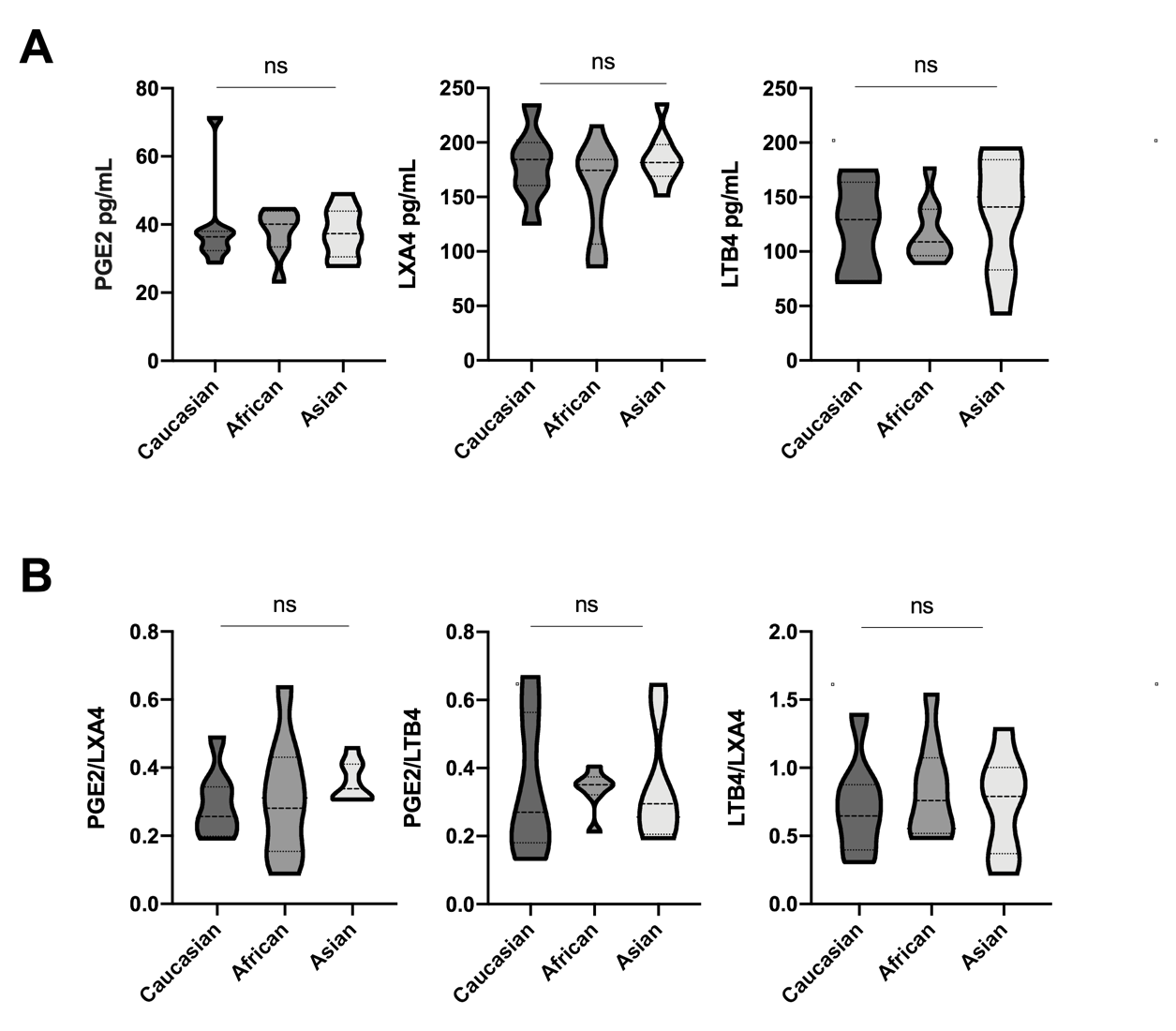

Supplement: Supplementary file 4 [file Image_4.tiff]

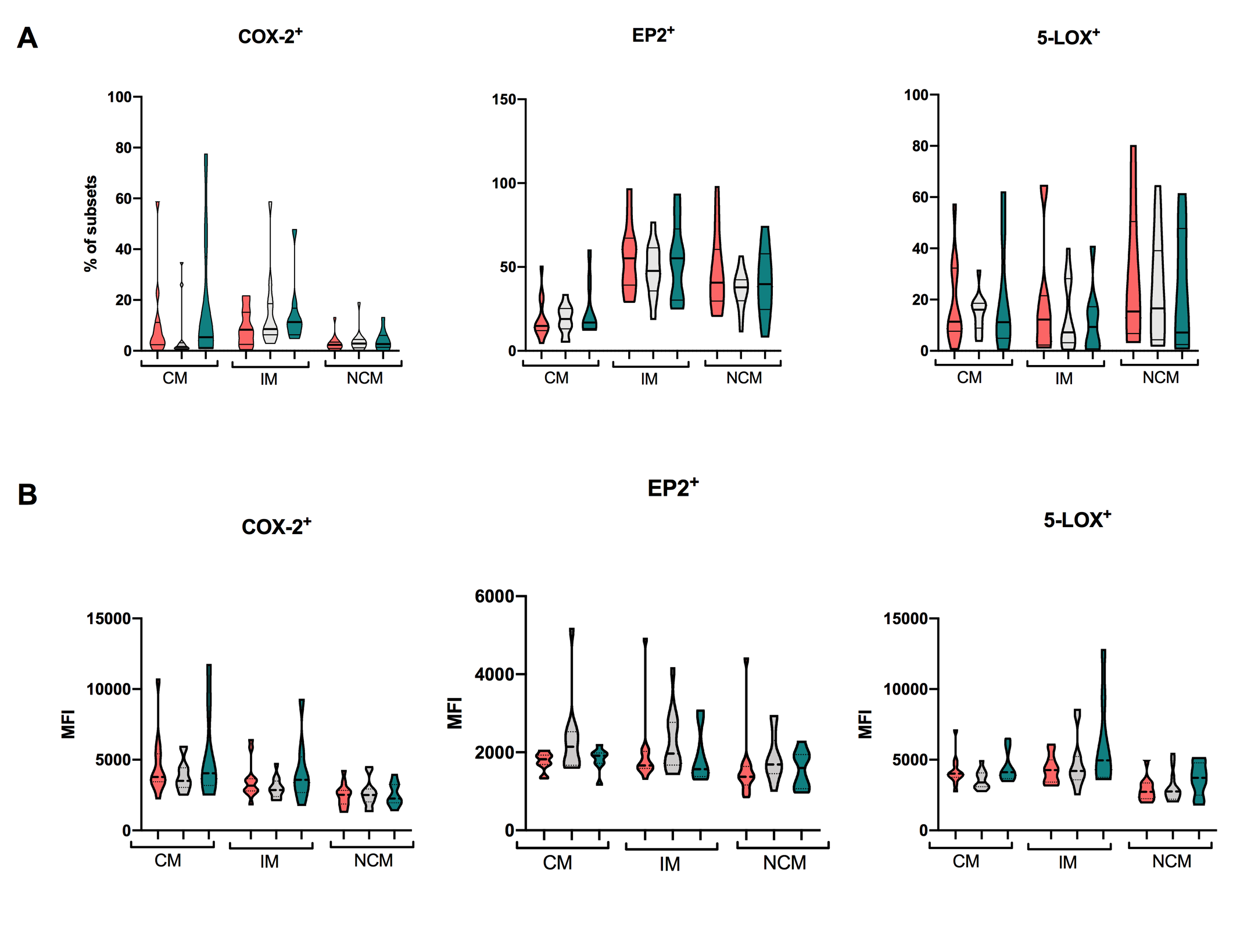

Supplement: Supplementary file 5 [file Image_5.tiff]

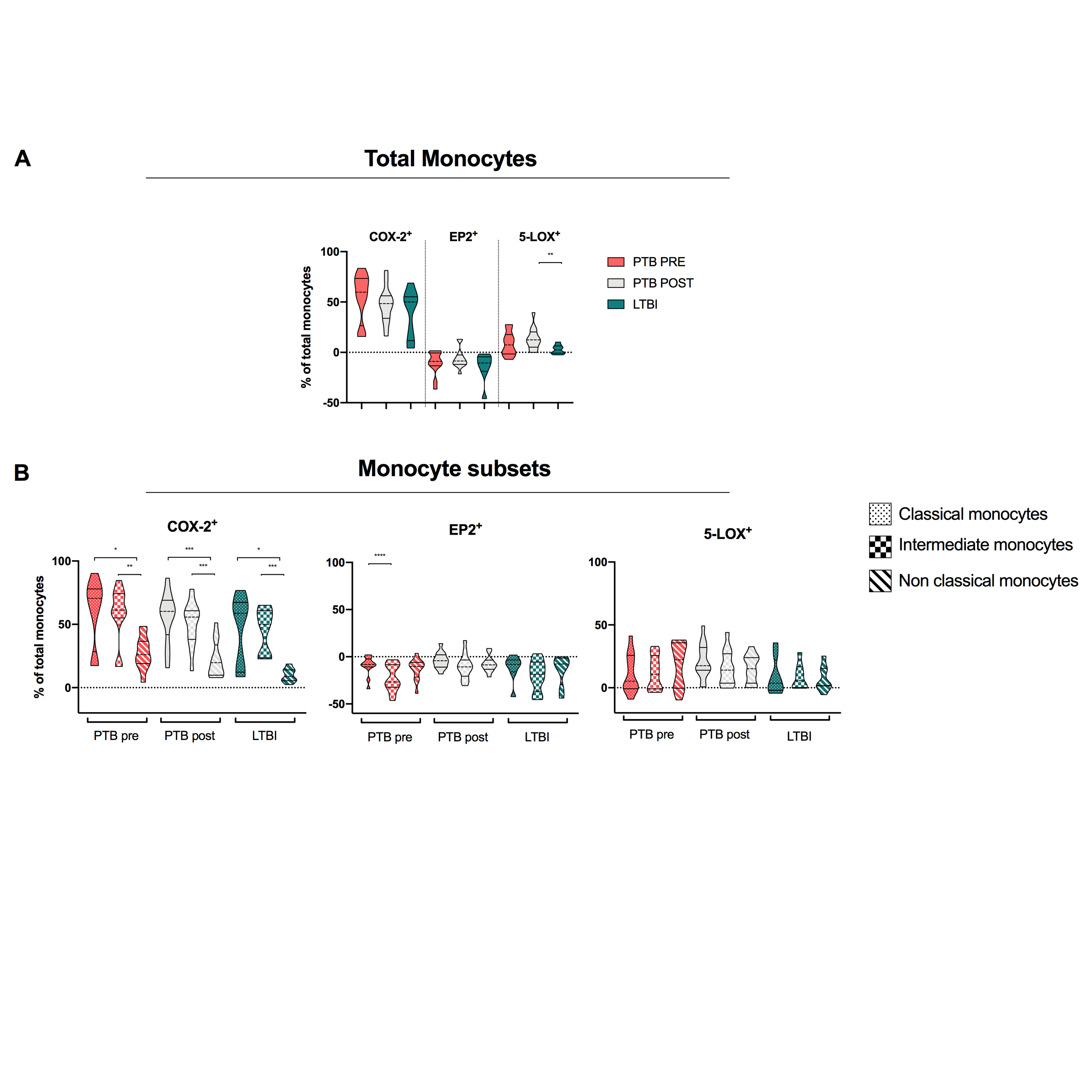

Supplement: Supplementary file 6 [file Image_6.tiff]

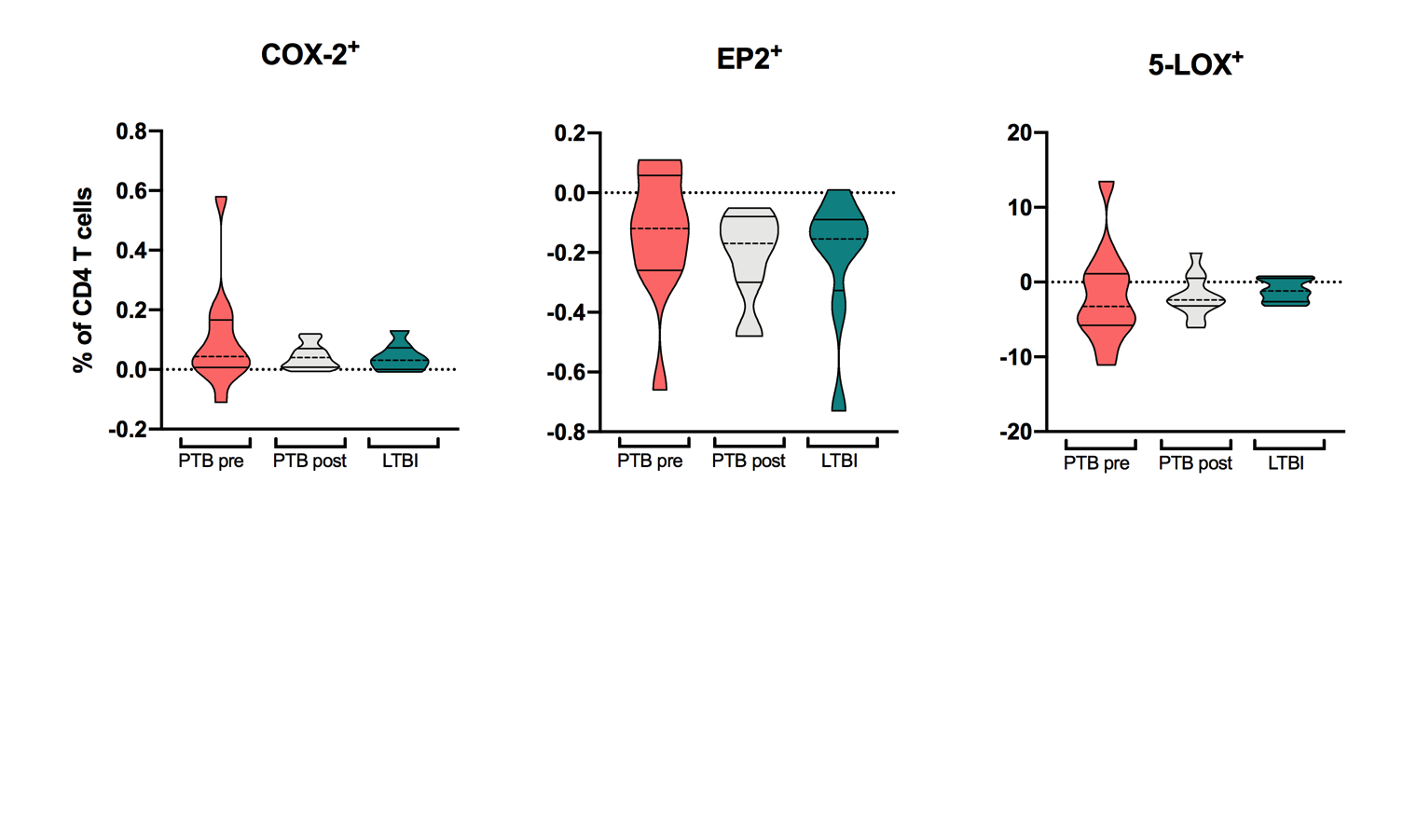

Supplement: Supplementary file 7 [file Image_7.tiff]
