# Supplementary material for: Elevated Levels of Anti-Inflammatory Eicosanoids and Monocyte Heterogeneity in Mycobacterium tuberculosis Infection and Disease
Source: Front Immunol. 2020 Nov 12;11:579849. doi: 10.3389/fimmu.2020.579849 (PMC7693556; doi:10.3389/fimmu.2020.579849)
Supplement: Supplementary file 8 [file DataSheet_1.docx]

Supplementary Material

# Supplementary Figures

**Supplementary Figure 1: Gating strategy.** Gating strategy showing total HLA-DR^+^ monocytes and monocyte subsets: classical monocytes (CD14^++^CD16^-^), Intermediate monocytes (CD14^++^, CD16^+^) and non-classical monocytes (CD14^+^CD16^++^) and expression of 5-LOX, EP2 and COX-2.

**Supplementary Figure 2: Gating strategy.** Gating strategy showing CD3^+^ CD4 T cells and expression of 5-LOX, EP2 and COX-2.

**Supplementary Figure 3: Gating strategy** Gating strategy showing FMO gating of COX-2, EP2 and 5-LOX, and gating for secondary antibody control for indirect staining of 5-LOX, for monocytes and T cells.

**Supplementary Figure 4: Plasma levels of eicosanoids in active TB patients stratified by ethnic background** Plasma levels **(A)** and ratios **(B)** of eicosanoid metabolites in pg/mL measured by ELISA in active TB patients (PTB and EPTB) with different ethnic background: Caucasian ( N=9), African ( N=11) and Asian (N=8). P-values were calculated using the Kruskal-Wallis test with Dunn’s post-hoc for multiple comparisons. Statistical significance represented by asterisk: ns, not significant; *, p <0.05; **, p <0.01; ***, p<0.001.

**Supplementary Figure 5: Comparison of COX-2, EP2 and 5-LOX in monocyte subsets between different stages of *Mtb infection.*** Frequencies **(A)** and MFI **(B)** of COX-2, EP2 and 5-LOX in monocyte subsets between different clinical states of *Mtb* infection; PTB at time of diagnosis (pink, N= 13), PTB at end- of- treatment (grey, N=13) and LTBI (green, n = 8). Monocyte subsets were defined as classical monocytes (CD14^++^CD16^-^), Intermediate monocytes (CD14^++^CD16^+^) and non-classical monocytes (CD14^+^CD16^++^) (Gating strategy supplementary figure 1A). No significant differences between patient groups were found. Wilcoxon matched-pairs signed rank test was used for group to group comparison, Mann-Whitney U test was used for unpaired data. Statistical significance represented by asterisk: *, p <0.05; **, p <0.01; ***, p<0.001.

**Supplementary Figure 6: Expression of** **COX-2, EP2 and 5-LOX in stimulated monocytes. (A)** Frequency of COX, EP2 and 5LOX in PPD-stimulated (background subtracted) total monocytes from PTB pre (pink), PTB post (grey) and LTBI (green). **(B)** Frequency of COX, EP2 and 5LOX in PPD-stimulated (background subtracted) monocyte subsets from PTB pre (pink), PTB post (grey) and LTBI (green). Wilcoxon matched-pairs signed rank test was used for group to group comparison, Mann-Whitney U test was used for unpaired data. Statistical significance represented by asterisk: ns, not significant; *, p <0.05; **, p <0.01; ***, p<0.001.

**Supplementary Figure 7: Frequency of COX-2, EP2 and 5-LOX in stimulated CD4^+^ T cells.** Expression of COX-2, EP2 and 5-LOX in PPD-stimulated (background subtracted) T cells from PTB pre (pink), PTB post (grey) and LTBI (green). No statistically significant differences between patient groups were found. Wilcoxon matched-pairs signed rank test was used for group to group comparison, Mann-Whitney U test was used for unpaired data. Statistical significance represented by asterisk: ns, not significant; *, p <0.05; **, p <0.01; ***, p<0.001.

# Supplementary Tables

**Supplementary table 1 ∣** Monocyte antibody panel

| **Primary Antibody** | **Secondary Antibody** | **Fluorochrome** | **Prod/Cat#** | **LOT** | **µL/dil** |
| --- | --- | --- | --- | --- | --- |
| Fixable viability 660 |  | APC | Ebio/65-0864-18 | 1961348 | 1:8000 |
| Lineage cocktail 1 |  | APC | Biolegend/363601 | B246137 | 10 |
| HLA DR |  | AX700 | Ebio 56-9956-42 | 1981610 | 4 |
| CD16 |  | BV605 | BD 563172 | 7299770 | 1.25 |
| CD14 |  | PerCP | Biolegend/325632 | B260983 | 4 |
| EP2 |  | PE | Cayman/ 10477 | 04537401 | 1:50 (2 ul) |
| COX-2 |  | FITC | Thermo/MA1-12235 | TL2687951 | 2,5 |
| 5-LOX | X | Alexa 405 | Abcam 169755/175654 | GR31974298 | 1:130/1:8000 |

**Supplementary table 2 ∣** T cell antibody panel

| **Primary Antibody** | **Secondary Antibody** | **Fluorochrome** | **Prod/Cat#** | **LOT** | **µL/dil** |
| --- | --- | --- | --- | --- | --- |
| Fixable Viability dye 660 |  | APC | Ebio/65-0864-18 | 1961348 | 1:8000 |
| CD3 |  | PerCP | BD/345766 | 8220696 | 5 uL |
| CD4 |  | Ax700 | Biolegend/300526 | 300526 | 1 uL |
| EP2 |  | PE | Cayman/ 10477 | 04537401 | 1:50 (2 ul) |
| COX-2 |  | FITC | Thermo/MA1-12235 | TL2687951 | 2,5 |
| 5-LOX | X | Alexa 405 | Abcam 169755/175654 | GR31974298 | 1:130/1:4000 |
